# A quantitative PCR method to detect blood microRNAs associated with tumorigenesis in transgenic mice

**DOI:** 10.1186/1476-4598-7-74

**Published:** 2008-09-30

**Authors:** Alice C Fan, Marianna M Goldrick, Jennifer Ho, Yu Liang, Pavan Bachireddy, Dean W Felsher

**Affiliations:** 1 Stanford University, School of Medicine, Division of Oncology, Departments of Medicine and Pathology, CCSR building, Room 1120, 269 Campus Drive, 94305-5151 Stanford, CA, USA; 2 Ambion/Applied Biosystems Inc., 2130 Woodward Suite 200, Austin, TX 78744, USA; 3 Applied Biosystems Inc, Division of Molecular Cell Biology-Assay R&D, 850 Lincoln Centre Drive, Foster City, CA 94404, USA; 4 BIOO Scientific, 3913 Todd Lane, Suite 312, Austin, TX 78744, USA

## Abstract

MicroRNA (miRNA) dysregulation frequently occurs in cancer. Analysis of whole blood miRNA in tumor models has not been widely reported, but could potentially lead to novel assays for early detection and monitoring of cancer. To determine whether miRNAs associated with malignancy could be detected in the peripheral blood, we used real-time reverse transcriptase-PCR to determine miRNA profiles in whole blood obtained from transgenic mice with c-MYC-induced lymphoma, hepatocellular carcinoma and osteosarcoma. The PCR-based assays used in our studies require only 10 nanograms of total RNA, allowing serial mini-profiles (20 – 30 miRNAs) to be carried out on individual animals over time. Blood miRNAs were measured from mice at different stages of MYC-induced lymphomagenesis and regression. Unsupervised hierarchical clustering of the data identified specific miRNA expression profiles that correlated with tumor type and stage. The miRNAs found to be altered in the blood of mice with tumors frequently reverted to normal levels upon tumor regression. Our results suggest that specific changes in blood miRNA can be detected during tumorigenesis and tumor regression.

## Findings

Distinct miRNA profiles have been described for many cancers including hematologic and solid malignancies [1-12]. Many reports have shown that patterns of miRNA expression differ between normal and cancerous tissues [1-10,12-20]. Gene expression profiling of traditional mRNA targets in whole blood or fractionated leukocytes has also shown correlations with many types of both neoplastic and non-neoplastic human disease, for example renal cancer and Crohn's disease [21-30]. To investigate whether miRNA patterns in blood correlated with tumorigenesis, we measured by qRT-PCR a panel of miRNAs in MYC-induced transgenic models of tumorigenesis.

First, we developed a protocol optimized for collection, storage and shipping of whole mouse blood, RNA extraction from a small volume of stored sample, and qRT-PCR assays for mouse blood miRNA profiling. To enable blood to be collected from mice at different time points and stored so that total RNA extraction and miRNA quantitation could be batch analyzed, mouse blood was mixed with an RNA stabilizing reagent (RNA*later*^® ^Tissue Collection:RNA Stabilization Solution, Ambion), transported, and stored at -20 deg C. Total RNA extraction was subsequently performed using the Mouse RiboPure™ Blood kit (Ambion).

**Figure 1:**
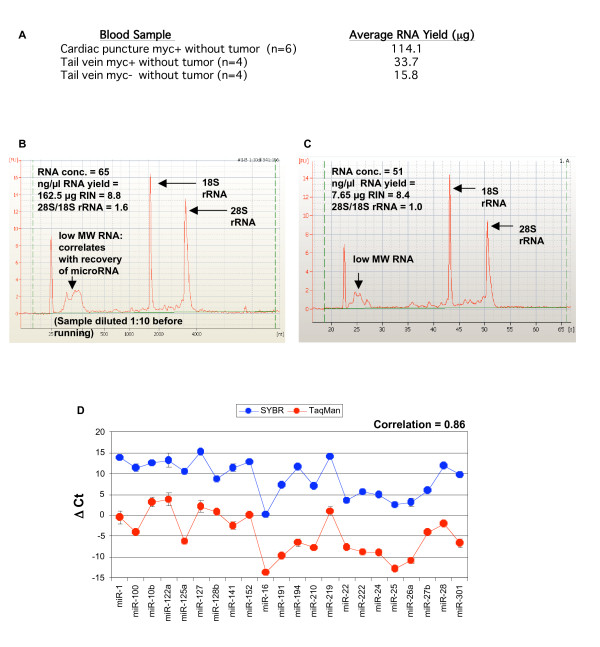
**High yields of intact RNA from stabilized mouse blood and use in qRT-PCR-based microRNA profiling**. **(A) **Yield of RNA isolated using the Ambion^® ^Mouse RiboPure™-Blood RNA Isolation kit. Blood samples were obtained by cardiac puncture or via tail vein bleed into a 2 mL tube preloaded with RNA*later*^® ^Solution. RNA was isolated, quantified, and examined on an Agilent^® ^2100 bioanalyzer (Agilent Technologies). Cardiac puncture samples were diluted 1:10 before running. Representative bioanalyzer scans for **(B) **Quality of cardiac puncture blood RNA and **(C) **Quality of tail vein blood RNA are shown with major features marked. The lack of significant species between the peaks indicates that RNA is highly intact. **(D) **Comparison of SYBR^®^-based and TaqMan^®^-based miRNA quantitation. A panel of 26 miRNA were quantitated in blood from normal control mice using both SYBR qRT-PCR assays and TaqMan-based qRT-PCR assays. Correlation between results from the two methods was 0.86.

Total RNA yields from cardiac puncture samples from 6 mice averaged 114 μg by NanoDrop measurement (Figure 1A). To determine the quality of total RNA extracted, Agilent bioanalyzer scans were performed. RNA was found to be intact as shown by the high RIN values observed in the Agilent traces and the sharp 18S and 28S ribosomal RNA (rRNA) peaks and lack of significant species between those peaks (Figure 1B, C). The bioanalyzer traces also showed a distinct peak that correlates with the recovery of low molecular weight RNA including miRNA. The high yields of RNA from mouse blood suggested that sufficient RNA for analysis might be extracted from lower volume tail vein bleeds that do not require sacrifice of the mouse. Indeed, blood acquired via tail vein from eight mice yielded an average of 15.8–33.7 μg RNA (Figure 1A). RNA yields from even the lowest amounts of tail vein blood were sufficient to run several hundred miRNA assays (as described below). Our initial experiments thus established the feasibility of carrying out blood miRNA profiling on individual animals during time-course experiments.

To determine which miRNAs could be readily detected in mouse blood, a panel of 111 quantitative reverse transcriptase PCR (qRT-PCR) SYBR miRNA assays was run on initial samples of blood from normal mice (see Additional file 1). The qRT-PCR assays were normalized via several methods, including 5S rRNA, U6 RNA, and global mean. Results were validated through the detection of several miRNA using TaqMan^® ^qRT-PCR assays. High concordance was observed in relative levels of blood miRNAs as determined by the SYBR-based and TaqMan-based assays, with a correlation value of 0.86 (Figure 1D). We conclude that qRT-PCR is a highly sensitive approach for identifying mouse blood miRNA profiles.

**Figure 2:**
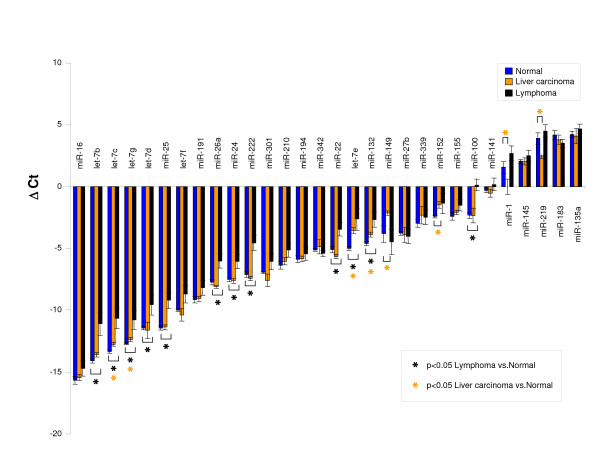
**(A) Comparison of relative blood miRNA profiles**. qRT-PCR results from 30 miRNA were compared in mice with MYC-induced lymphoma (black, n = 5), liver carcinoma (orange, n = 4) and healthy normal control mice without MYC overexpression (blue, n = 6). p-values < 0.05 for differences between liver carcinoma or lymphoma and normal individual miRNA are indicated with asterisks. MiRNA expression profiles were normalized with RNU6B. Mean ΔCt +/- standard error is graphed for each cohort.

To characterize blood miRNA patterns in mice with cancer, we utilized our previously described conditional transgenic mouse models of MYC-induced lymphoma, hepatocellular carcinoma and osteosarcoma [31-37]. 30 miRNAs that were reproducibly detectable using our methodology and known to be involved in the pathogenesis of cancer were selected for quantitation [5,14,16-18,20,38,39]. Significant differences were found in 12/30 miRNA in blood of mice with lymphoma, and 8/30 miRNA in blood of mice with liver tumors compared with normal controls (Figure 2). 25/30 miRNA for a mouse with osteosarcoma were also significantly different from normal controls, suggesting that further studies might be warranted in other tumor models (see Additional file 2). We conclude that changes in specific miRNAs can be detected in the blood of transgenic mice with lymphoma, hepatocellular carcinoma, and osteosarcoma. It is possible that we would have detected additional miRNA changes in mice with liver cancer if we had measured a broader panel of miRNAs. Alternatively, blood miRNA changes may be more readily detected in hematopoietic and mesenchymal tumors than in epithelial tumors.

**Figure 3:**
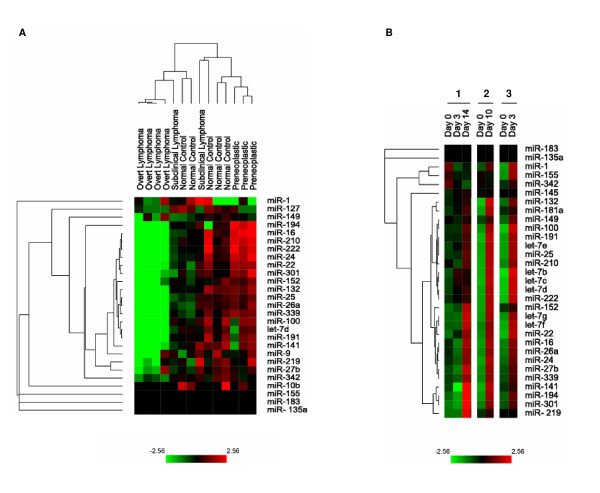
**(A) Hierarchical clustering of blood miRNA profiles in mice at different stages of tumor initiation and regression**. Blood samples were collected from normal mice without MYC overexpression (normal control, n = 5), mice with MYC activation prior to tumorigenesis (preneoplastic, n = 3), mice with early tumor formation (subclinical lymphoma, n = 2), and mice with large, clinically detectable lymphomas (overt lymphoma, n = 4). MiRNAs were grouped using an unsupervised hierarchical clustering approach and the results (fold change) are displayed with TreeView 1.6. **(B) **Changes in miRNA profiles during tumor regression. Serial blood samples were obtained by tail vein bleed from three mice with MYC-induced lymphoma before MYC inactivation (Day 0), and after different timepoints of MYC inactivation. MiRNA qRT-PCR results (fold change) are displayed with TreeView 1.6.

Next, we attempted to identify specific changes in miRNA profiles during lymphomagenesis. We performed qRT-PCR from blood specimens collected from transgenic mice before tumorigenesis (preneoplastic, n = 3), early tumor formation (subclinical lymphoma, n = 2) and after development of clinical lymphoma (overt lymphoma, n = 4). The results for a panel of 27 blood miRNA in all groups were compared to values from mice without MYC activation (normal control, n = 5) (Figure 3A). Unsupervised hierarchical clustering analysis revealed that overt lymphoma miRNA profiles grouped together. In mice with overt lymphoma, we identified a decrease in 20 miRNAs, including let-7d and miR-16 (Figure 3A). Notably, both of these miRNAs have been implicated in the pathogenesis of cancer [5]. Importantly, we found that the miRNA    profile in preneoplastic mice was markedly different from mice with overt lymphoma (Figure 3A). Hence, our results suggest the possibility that the performance of miRNA measurements in mice might be useful to monitor tumor development.

An important feature of our method is that its sensitivity should allow for the analysis of serial blood specimens from the same mouse. We obtained serial tail vein blood samples from mice with MYC-induced lymphoma before and after oncogene inactivation. The majority of miRNAs, including members of the let-7 family, were detectable at low levels and then increased upon tumor regression (Figure 3B) as early as 3 days after MYC inactivation even though tumors did not fully regress until after 14 days. Thus, changes in blood miRNA profiles can be determined from sequential peripheral blood samples drawn from individual mice.

An important caveat of our studies is that we cannot determine the source of the miRNA changes. A priori, we could be detecting miRNAs in rare circulating tumor cells or from host cells that are influenced by tumor growth. Regardless, the changes in miRNAs we see correlated with tumor progression, and thus may be useful as biomarkers of tumorigenesis.

Compared to microarray-based global miRNA profiling, the real-time PCR assays used in our studies require several magnitudes less input RNA and can be performed in substantially less time. These features of our method make it tractable for the detection of miRNA expression to identify early metastasis or minimal residual neoplastic cells. Moreover, we found that specific blood miRNA profiles mirrored specific types and states of cancer. Whether our approach will also be useful for the analysis of human patients remains to be determined. Finally, our method appears to be applicable to the sequential analysis of changes in miRNA expression in other models and in clinical materials. Our approach may be useful to identify biomarkers to detect early disease states and to predict clinical response.

## Materials and methods

### Transgenic mice

The TRE-MYC transgenic lines generated for these experiments were described previously [31-36,40-42]. The Eμ-tTA transgenic line for lymphoid specific expression and the Lt-tTA transgenic line for liver specific expression were both kindly provided by H. Bujard [43]. Oncogene expression was suppressed *in vivo *by injecting mice intraperitoneally with 20 μg of doxycycline in PBS and adding doxycycline (200 μg/ml) to the drinking water.

### Blood collection

Blood obtained by cardiac puncture from normal control mice of Balb/c and C57BL/6 strains was purchased from Jackson Labs (Bar Harbor MA). Blood from FVB/N and transgenic mice was collected at Stanford. To perform cardiac puncture, mice were euthanized using carbon dioxide, then the ventricle was accessed with an 18 gauge needle and 400–500 μl blood was aspirated into a 2 ml syringe. Blood was immediately discharged into a 2 ml microfuge tube preloaded with 1.3 ml RNA*later*^® ^Tissue Collection:RNA Stabilization Solution (Ambion, Austin TX), mixed by inversion, and stored at -20°C. To perform blood collection by tail vein, mice were placed under a heat lamp for 5 minutes, then a small peripheral tail incision was made. 2–10 drops of blood were collected directly into a 2 ml microfuge tube preloaded with 1.3 ml RNA*Later*^® ^Solution, mixed by inversion, and stored at -20°C. An average drop of blood was determined to be 24 μl. Blood/RNA*later*^® ^Solution mixtures were shipped on wet ice to a second site lab for RNA extraction and analysis.

### RNA extraction and analysis

The Ambion Mouse RiboPure™-Blood RNA Isolation kit (AB cat #AM1951) was used for extraction of RNA. Briefly, samples were centrifuged and the RNA*later*^® ^Solution removed prior to disruption of the blood pellet in a guanidinium-based lysis solution, followed by organic extraction and purification of the total RNA fraction (including small RNA) by solid phase extraction onto a silica matrix. The Alternative Protocol described in the kit instruction manual for samples less than 250 ul was used for extraction of RNA from the tail vein samples. RNA yields were determined by UV absorbance using a Nanodrop instrument (ND-1000 Spectrophotometer, NanoDrop Technologies) and intactness was examined on an Agilent^® ^2100 bioanalyzer (Agilent Technologies). The cardiac puncture samples were diluted 1:10 before running.

MiRNA analysis was carried out using the *mir*Vana qRT-PCR primer sets (Ambion) and the TaqMan^® ^MicroRNA Assays (Applied Biosystems)(26). The *mir*Vana qRT-PCR assays used 10 ng of input total RNA that was analyzed using target-specific primers for reverse transcription with M-MLV reverse transcriptase, followed by PCR amplification with a pair of miRNA target-specific primers and detection with SYBR^® ^Green I nucleic acid gel stain 10,000× concentrate in DMSO (Invitrogen). Melting curve analysis was carried out for each target to assess amplification specificity; for some targets, non-specific amplification was observed in the no-template negative controls, which could not be discriminated by melt-curve analysis. The TaqMan MicroRNA Assays used 10 ng of input total RNA with miRNA-target-specific reverse transcription primers and target-specific internal hybridization probes ("TaqMan probes"), and were run in 96-well or 384-well formats. qRT-PCR assays of similar design (also purchased from Applied Biosystems) were carried out for constitutively expressed small RNAs of similar size to miRNAs (e.g. snoRNAs) and used for normalization of input RNA amount (analogous to use of constitutive mRNAs such as GAPDH for normalization of protein-coding genes).

The reverse transcription reactions were carried out for 65 min and used the AB TaqMan^® ^microRNA Reverse Transcription Kit (AB cat #4366597) which includes M-MLV reverse transcriptase. Amplification reactions consisted of a hold of 10 min at 95°C and 40 cycles (15 sec/95°C, 60 sec/60°C) on an Applied Biosystems 7900HT Real-Time PCR System and required about 1.5 hours to complete. The assays were carried out in duplicate or triplicate and the geometric average Ct value was used to calculate relative expression for each datapoint. Unsupervised hierarchical clustering of samples was carried out with the program Cluster 3.0. Each sample was used in multiple experimental runs, and relative expression of different miRNAs was determined using identical endogenous controls in each experiment. Within each experiment, the endogenous control that had the highest Ct was set as the baseline, and the Ct between the baseline and the Ct of the small RNA control in each sample was used as a normalization factor that was added to the raw Ct for each sample. Normalized Ct values larger than 35 were reported as 35. After mean-centering the data for each miRNA and using uncentered correlation similarity metric and average linkage, the expression of miRNAs was hierarchically clustered and displayed with the TreeView 1.6.

## Competing interests

At the time the experiments were performed, Marianna Goldrick, Jennifer Ho and Yu Liang were scientists employed by Ambion/Applied Biosystems.

## Authors' contributions

ACF, MMG and DWF conceived and designed the experiments. ACF, MMG, JH and PB performed experiments. MMG, JH, and YL analyzed data. ACF, MMG, PB and DWF wrote the manuscript. All authors read and approved the final manuscript.

## Supplementary Material

Additional file 1 qRT-PCR results from 111 miRNA in blood from normal mice. Bar graphs of qRT-PCR results from 111 miRNA in blood from normal mice, normalized with 5S. Mean ΔCt +/- standard error is graphed for each miRNA. Click here for file

Additional file 2 Blood miRNA expression associated with MYC-induced osteosarcoma. Blood miRNA expression in mouse with MYC-induced osteosarcoma (orange, n=1) and healthy control mice without MYC overexpression (blue, n = 6) were normalized with RNU6B. Mean ΔCt +/- standard error is graphed for each miRNA. Click here for file
