# Supplementary figures and images for: A quantitative PCR method to detect blood microRNAs associated with tumorigenesis in transgenic mice
Source: Mol Cancer. 2008 Sep 30;7:74. doi: 10.1186/1476-4598-7-74 (PMC2572631; doi:10.1186/1476-4598-7-74)

$\Delta Ct$

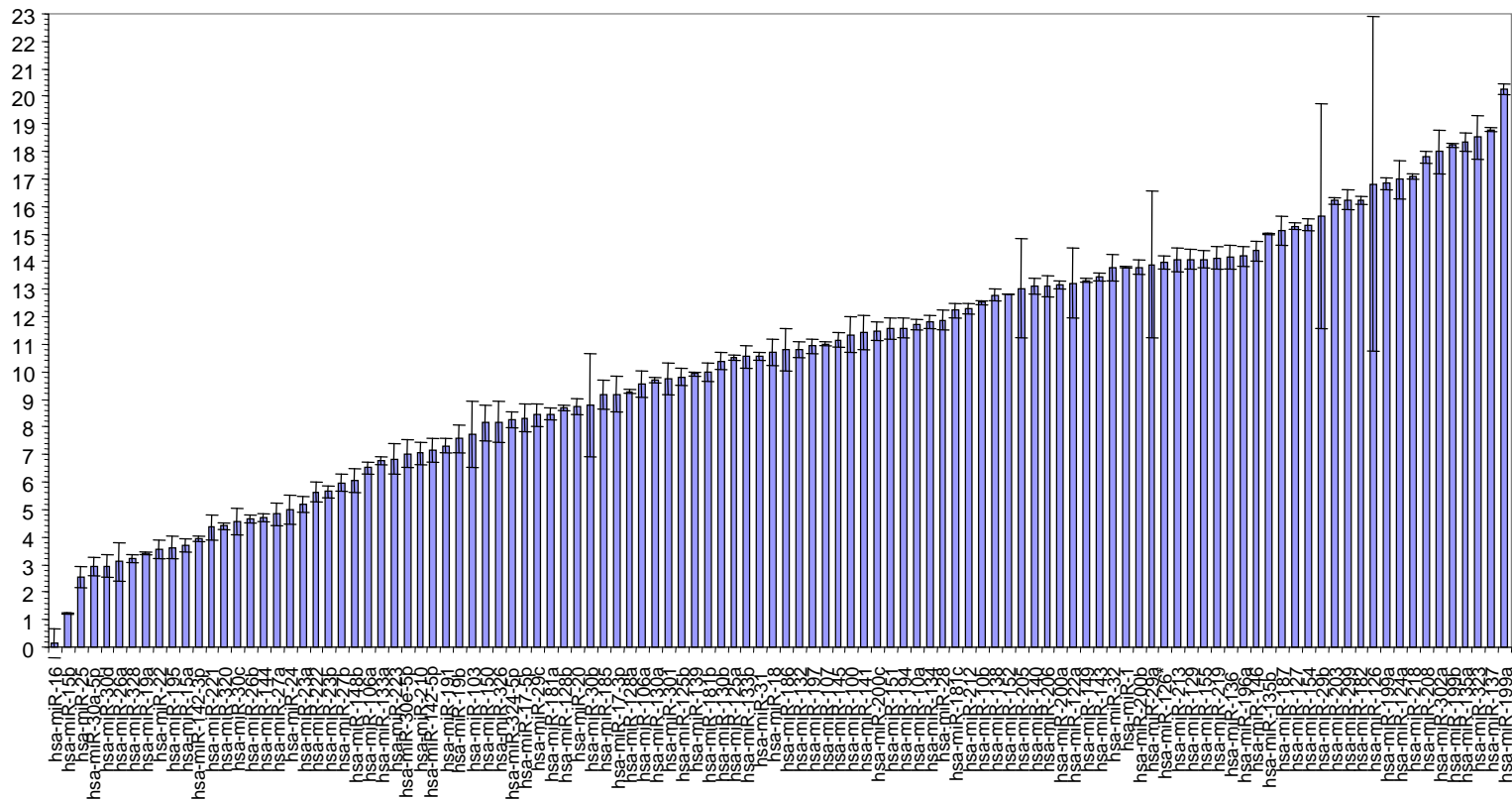

MiRNA

Supplement: Additional file 1 — qRT-PCR results from 111 miRNA in blood from normal mice. Bar graphs of qRT-PCR results from 111 miRNA in blood from normal mice, normalized with 5S. Mean ΔCt +/- standard error is graphed for each miRNA. [file 1476-4598-7-74-S1.pdf]

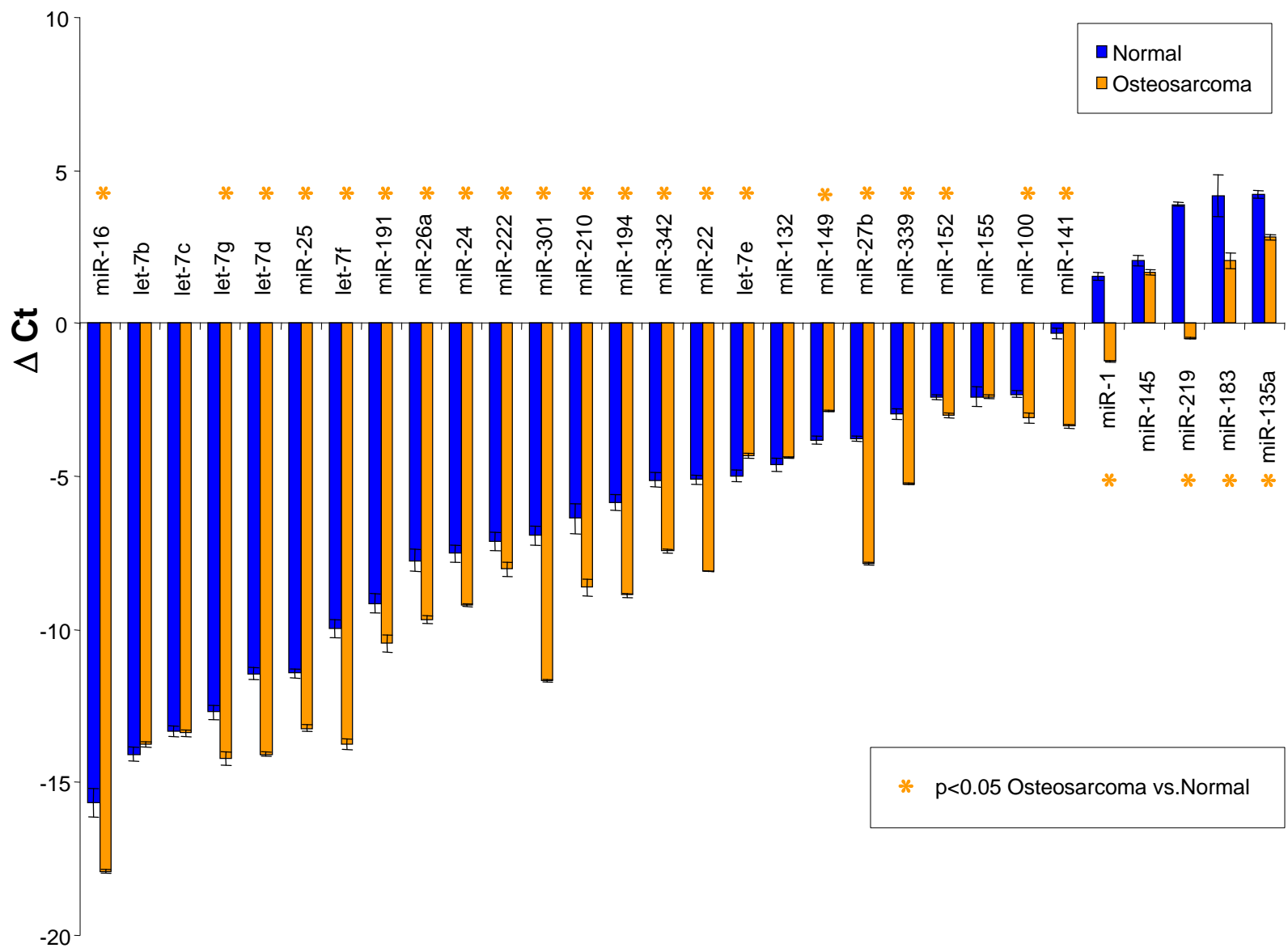

Supplement: Additional file 2 — Blood miRNA expression associated with MYC-induced osteosarcoma. Blood miRNA expression in mouse with MYC-induced osteosarcoma (orange, n=1) and healthy control mice without MYC overexpression (blue, n = 6) were normalized with RNU6B. Mean ΔCt +/- standard error is graphed for each miRNA. [file 1476-4598-7-74-S2.pdf]
